# Relationships Between Chemical Structure and Antioxidant Activity of Isolated Phytocompounds from Lemon Verbena

**DOI:** 10.3390/antiox8080324

**Published:** 2019-08-20

**Authors:** Noelia Sánchez-Marzo, Jesús Lozano-Sánchez, María de la Luz Cádiz-Gurrea, María Herranz-López, Vicente Micol, Antonio Segura-Carretero

**Affiliations:** 1 Instituto de Biología Molecular y Celular (IBMC) and Instituto de Investigación, Desarrollo e Innovación en Biotecnología Sanitaria de Elche (IDiBE), Universitas Miguel Hernández, 03202 Elche, Spain; 2 Department of Food Science and Nutrition, University of Granada, Campus of Cartuja, 18071 Granada, Spain; 3 Research and Development of Functional Food Centre (CIDAF), PTS Granada, Avda. Del Conocimiento s/n., Edificio BioRegion, 18016 Granada, Spain; 4 Department of Analytical Chemistry, University of Granada, C/Fuentenueva s/n, 18071 Granada, Spain; 5 CIBER: CB12/03/30038, Fisiopatología de la Obesidad y la Nutrición, CIBERobn, Instituto de Salud Carlos III (ISCIII), 07122 Palma de Mallorca, Spain

**Keywords:** lemon verbena, RP-HPLC-ESI-TOF-MS, semi-preparative chromatography, isolation, antioxidant, structure-activity relationship

## Abstract

Over the last few years, people have been concerned about the narrow relationship between nutrition and health leading to an increasing demand of nutraceutical products and functional food. Lemon verbena (*Lippia citriodora* Kunth) has been traditionally used for respiratory, digestive, and muscular diseases, showing effects that are promoted by the antioxidant activity of its phytoconstituents. The antioxidant power of several lemon verbena extracts has been tested but its isolated compounds activity has not been described. The aim of the present work was to isolate phytochemicals from a commercial lemon verbena extract through a semi-preparative high-performance liquid chromatography approach for further evaluation of its individual antioxidant activity using three different methods. The structure-antioxidant activity relationships revealed the influence of substitutions in the strong antioxidant power exerted by glycosylated phenylpropanoids, in contrast to the low antioxidant capacity showed by iridoids. Development of enriched extracts in these compounds could lead to greater antioxidant effects and improved functional ingredients to prevent chronic diseases.

## 1. Introduction

Combinatory chemistry has demonstrated to be insufficient for designing bioactive molecules and there is growing interest in discovering new compounds from natural sources. Plants have been used for centuries in traditional medicine, so they constitute a very attractive source for elucidating new structures that could interact with human biomolecules.

One well-known medicinal plant is lemon verbena, with scientific names of *Lippia citriodora* (Kunth), *Aloysia citriodora* (Paláu), and *Aloysia triphylla* (L’Hérit). It belongs to the family Verbenaceae (Lamiales order) and is native from South America but is also cultivated in southern Europe and northern Africa. Lemon verbena was widely employed for stomach and nervous disorders by the Inca culture and there are reports of this specie from the 17th century [1]. Several studies have been reported its beneficial effects, including antimicrobial, neuroprotective, cardioprotective, anticonvulsant, anti-inflammatory, and antigenotoxic among others recently reviewed by Bahramsoltani et al. [2]. The biological activity of this plant has been demonstrated in vitro and in vivo [3,4]. Furthermore, eight clinical trials have been carried out using lemon verbena extracts [5,6,7,8,9,10,11,12]. Most of them lasted 21–28 days and no adverse effects were reported, showing the safety of those extracts. Four of the clinical studies were related to the antioxidant power of lemon verbena, increasing the activity of antioxidant enzymes such as glutathione reductase and glutathione peroxidase while oxidation markers in plasma, i.e., protein carbonyls and malondialdehyde, were diminished [6,7,8,9].

Oxidative stress is involved in important chronic disorders such as neurodegenerative diseases, obesity, and cancer. Reactive oxygen species (ROS) are the most studied components of oxidative stress and can damage proteins, lipids, and DNA [13]. ROS not only cause DNA oxidation products but can also affect DNA repair proteins, compromising their efficiency and leading in carcinogenesis [14]. Furthermore, the overproduction of ROS alters important metabolic pathways and induces the expression of inflammatory mediators [15]. In this context, antioxidants could prevent the development of chronic conditions. Lemon verbena has shown its antioxidant potential as it has been tested on an insulin-resistant hypertrophic 3T3-L1-adipocyte model and colon cancer cells, exhibiting promising results [2,16].

Various categories of phytochemicals have been described in different parts of *L. citriodora*, including terpenoids and phenolic compounds, which are the most relevant groups of bioactive compounds [17]. Fatty alcohols and ketones have also been identified [18,19]. Verbascoside, also named acteoside, a polyphenol from the phenylpropanoids subgroup, is the most abundant compound in lemon verbena leaves so that the related biological effects are mainly attributed to this phytochemical. The antioxidant power of isolated verbascoside has been extensively studied [20,21,22]. Nevertheless, no data are available on the antioxidant activity of other compounds from lemon verbena that could be responsible of the observed effects.

Due to the lack of commercial standards, isolating compounds from original extracts is necessary for studying the bioactivity on their own and exploring structure-activity relationships. One of the most efficient techniques to fractionate extracts is (semi-)preparative liquid chromatography (LC), and C18 reversed phase (RP) columns offer high versatility to purify terpenoids and phenolic compounds [23]. C18-RP columns are also employed to characterize the composition of whole extracts and collected fractions by high-pressure liquid chromatography (HPLC). This separation methodology can be coupled to different detection systems and constitute a very useful analytical tool. Ultraviolet and diodo-array detectors (DAD) allow the detection of phenolic compounds but mass spectrometry (MS) provides information about their molecular weight and structure [24]. Moreover, the most probable molecular formula can be deduced through MS with a time-of-flight (TOF) analyzer from accurate mass data and its elevated sensitivity [25].

There are well-established methods to examine the antioxidant capacity of whole extracts and isolated compounds in vitro [26]. They can differ in the mechanism of reduction of generated oxidant species by the assessed compounds, and single electron transfer (SET) methods can be distinguished from those based on hydrogen atom transfer (HAT).

Hence, the aims of the present study were: (1) To characterize the composition of a commercial lemon verbena extract using HPLC coupled to a TOF mass spectrometer with an electrospray ionization (ESI) interface, (2) to obtain different fractions from the extract by semi-preparative HPLC and to analyze their composition by HPLC-ESI-TOF-MS, and (3) to examine the antioxidant activity of the whole extract and the fractions by three different methods for clarifying structure-activity relationships.

## 2. Materials and Methods

### 2.1. Chemicals

Acetic acid and methanol were purchased from Fluka (Sigma-Aldrich, Steinheim, Germany) and Lab-Scan (Gliwice, Poland), respectively. Both solvents were of HPLC-MS grade and the water for all experiments was purified by a Milli-Q system from Millipore (Bedford, MA, USA). For the antioxidant assays, ABTS [2,2′-azinobis (3-ethylbenzothiazoline-6-sulfonate)], Trolox (6-hydroxy-2,5,7,8-tetramethylchroman-2-carboxylic acid), AAPH (2,2′-azobis-2-methyl-propanimidamide, dihydrochloride), TPTZ (2,4,6-tripyridyl-S-triazine), ferric sulfate, potassium persulfate and fluorescein were obtained from Sigma-Aldrich (St. Louis, MO, USA). The rest of reagents needed for measuring the antioxidant activity were provided from Panreac (Barcelona, Spain): Ferric chloride, hydrochloric acid, sodium acetate, trihydrated sodium acetate and dehydrated sodium phosphate.

### 2.2. Sample Preparation

A commercial extract (PLX^®^10) of *Lippia citriodora* was used in this work containing 10% verbascoside, as declared by the manufacturer (Monteloeder, Elche, Spain).

For analytical characterization, 5 mg of extract were dissolved in 1 mL of water. After 1 min of vortex and sonication for 20 s, the result solution was filtered through a 0.25 mm filter before the HPLC analysis. Meanwhile, a solution of lemon verbena extract at 50 mg/mL was prepared in water for its fractionation by semi-preparative HPLC. This solution was vortexed for 2 min, was sonicated for 20 s and then was filtered employing the mentioned filter.

### 2.3. Instrumentation

Different fractions of the *L. citriodora* extract were recovered using a Gilson preparative HPLC system (Gilson Inc., Middleton, WI, USA) which equipped with automated liquid handling solutions (model GX-271), a binary pump (model 331/332) and UV-vis detector (model UV-Vis 156). The solvent of the collected fractions was evaporated in a Savant SpeedVac Concentrator SC250 EXP (Thermo Scientific, Waltham, MA, USA).

The composition of the whole extract and the fractions was analyzed through a HPLC-ESI-TOF-MS system. The chromatographic separation was carried out using an Agilent 1200 series rapid-solution LC equipment (Agilent Technologies, Palo Alto, CA, USA) with an autosampler, a binary pump and a diode-array detector (DAD).

Both HPLC systems were coupled to a time-of-flight (TOF) mass spectrometer (Bruker Daltonics, Bremen, Germany) with an electrospray ionization (ESI) interface (model G1607A, Agilent Technologies, Palo Alto, CA, USA). Calibration solution was injected at the beginning of each analysis by a 74900-00-05 Cole-Palmer syringe pump (Cole-Palmer, Vernon Hills, IL, USA) directly connected to the interface.

Finally, absorbance and fluorescence measurements for the antioxidant assays were performed on a Synergy Mx Monochromator-Based Multi-Mode Micro plate reader (Bio-Tek instruments Inc., Winooski, VT, USA) in 96-well microplates.

### 2.4. Chromatographic and UV Conditions

The separation of the compounds present in the lemon verbena extract and the collected fractions was performed at room temperature using a Zorbax Eclipse Plus C18 column (1.8 µm, 150 × 4.6 mm). The mobile phase consisted of acetic acid 0.5% in water as eluent A and methanol as eluent B. The flow rate was set at 0.4 mL/min and the following multi-step linear gradient was employed: 0 min, 5% B; 3 min, 10% B; 5 min, 14% B; 15 min, 20% B; 17 min, 23% B; 29 min, 35% B; 36 min, 38% B; 52 min, 60% B; 54 min, 95% B; 56 min, 5% B; 65 min, 5% B. The initial conditions were maintained for 10 min and the injection volume was 10 µL. The spectrum range of 190–950 nm was monitored by the DAD detector.

### 2.5. Fractionation of Phytochemicals

The lemon verbena extract was fractionated with the aim of obtaining isolated compounds. The separation was carried out using a semi-preparative Ascentis C18 column (10 µm, 250 × 212 mm) at room temperature. The mobile phase used was the same than for the analytical characterization, acetic acid 0.5% (A) and methanol (B), and the following multi-step linear gradient was applied: 0 min, 5% B; 15 min, 20% B; 21 min, 23% B; 28 min, 25% B; 32 min, 32% B; 52 min, 40% B, 69 min, 60% B; 71 min, 65% B; 75 min, 100% B; 80 min, 5% B; 84 min, 5% B. In this case, the flow rate used was 10 mL/min and the injected volume was 500 µL. The UV-vis detector of the Gilson preparative HPLC system was set at 240 and 280 nm. The fraction collection step consisted of mass spectrometry (MS)-based purification, determining the elution time window for collecting the target compound. A total of 19 fractions were obtained and their solvent was evaporated at 35 °C under vacuum. The residue of each fraction was dissolved in water at a final concentration of 1 mg/mL. The result solutions were filtered through a 0.25 mm filter and were stored at –20 °C until their characterization and further antioxidant evaluation.

### 2.6. ESI-TOF-MS Detection

HPLC systems were coupled to a TOF mass spectrometer in which a stable spray was ensured with a flow of 0.2 mL/min. For that reason, a splitter for both, analytical, and semipreparative HPLC systems, was used in the coupling with the MS detector (make-up pump equipped with a MRA splitter, model 307, Gilson, Middleton, WI, USA).

The ESI interface operated in negative ion mode, setting a capillary voltage of +4.5 kV and other source parameters were optimized. Briefly, the drying gas temperature and flow were 210 °C and 9 L/min, respectively, and nebulizing gas pressure was 2.3 bar. The following values were set concerning the transfer parameters: Capillary exit, −120 V; skimmer 1, −40 V; skimmer 2, −22.5 V; hexapole 1, −23 V; hexapole 2, −20 V.

Data were acquired considering a *m/z* range of 50–1000. The accurate mass data of the molecular ions were processed through the software DataAnalysis 4.0 (Bruker Daltonics, Bremen, Germany) and its Generate Molecular Formula Editor provided possible elemental formulas with high confidence through comparison of the theorical and the measured isotope pattern (σ value). The accuracy threshold was established at 5 ppm as it is widely accepted [27]. All spectra were calibrated prior to the compound identification through a sodium formate cluster as calibration solution, containing 5 mM sodium hydroxide and 0.2% acetic acid in water:isopropanol 1:1 v/v, injected at the beginning of each run.

### 2.7. Antioxidant Activity Assays

The antioxidant activity of the lemon verbena extract and the obtained fractions was evaluated. Two single-electron transfer (SET) based methods were developed—the ferric ion reducing antioxidant power (FRAP) and the trolox equivalent antioxidant capacity (TEAC) assays—the while oxygen radical absorbance capacity (ORAC) method was also performed as a hydrogen-atom transfer (HAT) based test.

#### 2.7.1. Ferric-Reducing Ability Power Assay (FRAP)

The FRAP assay was carried out following the method described by Benzie and Strain [28]. Reduction of a ferric-tripyridyltriazine complex was estimated mixing the samples with freshly prepared FRAP reagent and measuring the absorbance at 593 nm for 4 min. A standard curve of FeSO_4_·7H_2_O was assessed and results were expressed as millimoles of Fe^2+^ equivalents (FE) per gram of extract or fraction (dry weight, dw).

#### 2.7.2. Trolox Equivalent Antioxidant Capacity (TEAC)

The TEAC assay was originally described by Miller et al. and was performed as modified by Cádiz-Gurrea et al. [29,30]. This method consists of the ABTS [2,2′-azinobis-(3-ethylbenzothiazoline-6-sulphonate)] radical cation (ABTS^+•^) scavenging activity of the samples. Briefly, ABTS stock solution was prepared with 2.45 mM potassium persulfate to generate the radical cation specie. After 12–24 h in darkness at room temperature, the absorbance value of the resulting ABTS^+•^ solution was adjusted to 0.70 (± 0.02) at 734 nm through water dilution prior its use. Samples were mixed with this solution and runs were performed at 734 nm and 25 °C after 5 min of reaction. A standard curve of trolox was prepared for expressing the antioxidant activities as millimoles of trolox equivalents (TE) per gram of extract or fraction (dw).

#### 2.7.3. Oxygen Radical Absorbance Capacity (ORAC)

The ORAC method developed by Ou el al. was performed to assay the capacity of the samples to scavenge peroxyl radicals with some modifications as Cádiz-Gurrea et al. reported [30,31]. A freshly prepared AAPH solution was used for generating peroxyl radicals and fluorescein oxidation was monitored at 37 °C with 495 nm excitation and 520 nm emission filters. In this case, trolox was also used as reference compound and a regression equation between its concentration and the net area of the fluorescence decay curve (area under curve, AUC) was employed to calculate the final ORAC values of the samples expressed as mmol TE/g (dw).

## 3. Results and Discussion

### 3.1. Characterization of A L. citriodora Extract by HPLC-ESI-TOF-MS

**Figure 1:**
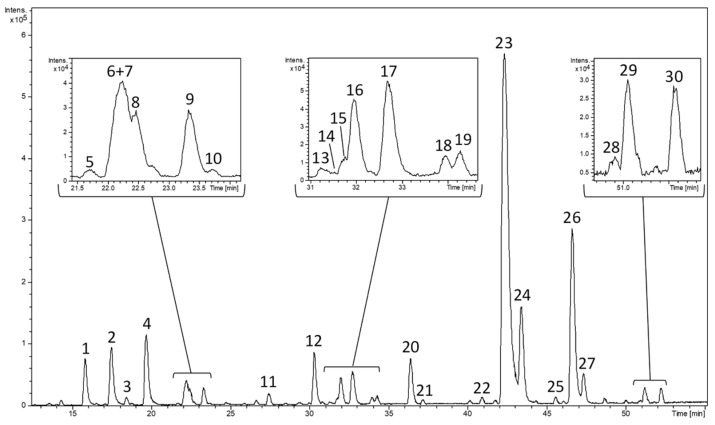
Base peak chromatogram (BPC) of a commercial lemon verbena extract (PLX^®^10) at 5 mg/mL obtained by high-pressure liquid chromatography (HPLC)-electrospray ionization interface (ESI)-time-of-flight (TOF)-mass spectrometry (MS) in negative ion mode. Peak numbers correspond to those of Table 1 according to the elution order.

A commercial *L. citriodora* was characterized through mass spectrometry detection after chromatographic separation of its constituents with a reversed phase. Figure 1 shows the base peak chromatogram (BPC) corresponding to negative polarity. A total of 30 compounds were tentatively identified and were numbered according to their elution order.

Table 1 includes the information provided by the analytical system including the retention time, the mass spectral data (experimental and calculated *m/z*, mass error, and σ values) and the molecular formula predicted by the software for each peak. The elution order, UV-vis spectra, MS data, and information provided from literature were taken into consideration to assign the proposed compounds. Iridoids, glycosylated phenylpropanoids, and flavonoids were mainly found.

#### 3.1.1. Iridoids

Most compounds that were eluted firstly were characterized as iridoids. Shanziside (*m/z* 391), gardoside (*m/z* 373), ixoside (*m/z* 387) and theveside (*m/z* 389) were tentatively identified for peaks 1–3 and 12, respectively. These compounds were previously described in *L. citriodora* by other authors who considered the fragmentation patterns (MS/MS) [17,32].

Furthermore, not only loganic acid (peak 9) but also epiloganic acid (peak 8) and secologanic acid (peak 10) were found. Loganic acid (*m/z* 375) had been previously reported in *L. citriodora* but, as far as we are concerned, epiloganic (*m/z* 375) and secologanic (*m/z* 373) acids have never been described in this specie [33]. Epiloganic and secologanic acids were characterized in *Lippia graveolens* leaves using nuclear magnetic resonance spectroscopy (NMR) [34]. Both compounds were tentatively proposed on basis of the data provided by the mass spectrometer and their elution order was corroborated with another study [35].

The peak 11 was identified as shanziside methyl ester (*m/z* 405) which has been found in different species from *Lippia* genus as *L. citriodora* and *L. alba* [33,36]. For peak 13, teucardoside (*m/z* 489) was tentatively postulated on basis of its presence in other species from Lamiales order as other authors also indicated in a previous report [37].

#### 3.1.2. Glycosylated Phenylpropanoids

A total of ten compounds were identified as glycosylated phenylpropanoids, which constitute an abundant group of phenolic compounds in lemon verbena as it is described in the bibliography [32]. The most intense peak (peak 23) was proposed as verbascoside and the second most intense one (peak 26) was associated with its isomer isoverbascoside, presenting both *m/z* values at 623. Their β-hydroxyverbascoside and β-hydroxyisoverbascoside derivatives were postulated for peaks 18/19 (*m/z* 639). Their identical mass spectra and close elution time did not allow their particular identification in our work. Indeed, the fragmentation pattern of these two derivatives generates identical fragments which did not allow their distinction through MS/MS in a previous characterization of *L. citriodora* [17]. In that study, verbasoside (*m/z* 461) and martynoside (*m/z* 651) were also described corresponding to peaks 4 and 30 of the present work, respectively.

The same molecular formula was predicted for peaks 6 and 7. Both compounds were postulated as cistanoside F isomers (*m/z* 487) according to the literature [33,38]. The peak 27 was identified as leucosceptoside A (*m/z* 637) which has been reported in *L. citriodora*, *L. alba* and *L. multiflora* [39,40,41].

In contrast, isoleucosceptoside A (*m/z* 637), corresponding to peak 28, has been tentatively proposed for the first time in the *Lippia* genus. This compound, also named plantanoside C, was characterized using NMR in Citharexylum spinosum which also belongs to Verbenaceae family [42]. The elution order of isoleucosceptoside A was consistent with another study using *Globularia* spp. (Lamiales order) where other common compounds were also described (gardoside, verbascoside, and leucosceptoside A) [43]. A maximum absorbance of 280 nm was observed for this peak corroborating its phenolic structure (Supplementary Figure S1).

#### 3.1.3. Flavonoids

Four flavonoids belonging to flavones subclass were identified in the lemon verbena extract, as were described in other works [17,32]. These flavones were luteolin-7-diglucuronide (peak 20, *m/z* 637), apigenin-7-diglucuronide (peak 22, *m/z* 621), chrysoeriol-7-diglucuronide (peak 24, *m/z* 651), and acacetin-7-diglucuronide (peak 29, *m/z* 635). Glucuronidation occurs in plants for enhancing the flavonoid solubility in water and favoring its accumulation in vacuoles [44]. Moreover, diooflavone (*m/z* 621) that is a biflavonoid was also detected (peak 25) in the present work as occurred in other lemon verbena extract characterization [33].

#### 3.1.4. Other Compounds

Three monoterpenoids were found in their glycosylated form, sacranoside A (peak 15, *m/z* 445) and two tuberonic acid glucoside isomers (peaks 14 and 16, *m/z* 387), that have been described in bibliography related to *L. citriodora* [33]. Peak 21 was tentatively proposed lariciresinol-4-O-β-D-glucopyranoside (*m/z* 521) which belongs to lignans and has been identified in *L. graveolens* through NMR and circular dichroism [45].

Furthermore, the molecular formula generated for peak 5 (*m/z* 299) and the literature allowed for its tentative identification as salidroside for the first time in this specie. Salidroside is a tyrosol (simple phenol) derivative which is present in some lamial species [46,47,48]. Peak 5 presented an absorbance maximum at 280 nm that is the maximum wavelength of absorption observed in tyrosol and its derivatives (Supplementary Figure S1).

Nevertheless, retention time, UV absorption, and mass spectra were not sufficient for the identification of peak 17 (*m/z* 373), contrasting with literature and databases. Additional information on fragmentations in MS/MS experiments is required for their elucidation.

### 3.2. Fractionation of the Lemon Verbena Extract Using Semi-Preparative Chromatography

The compounds in the lemon verbena extract were isolated through a semi-preparative HPLC methodology Previously, the optimization of the developed analytical method was required in the scaled-up process. Columns with higher particle size are used in (semi-) preparative chromatography. This fact causes lower peak resolution but its bigger capacity leads to a higher amount of compound obtained per unit time. On the other hand, longer columns provide greater resolution [49]. For these reasons, the optimization of the semi-preparative method was required in the scaled-up process.

**Figure 2:**
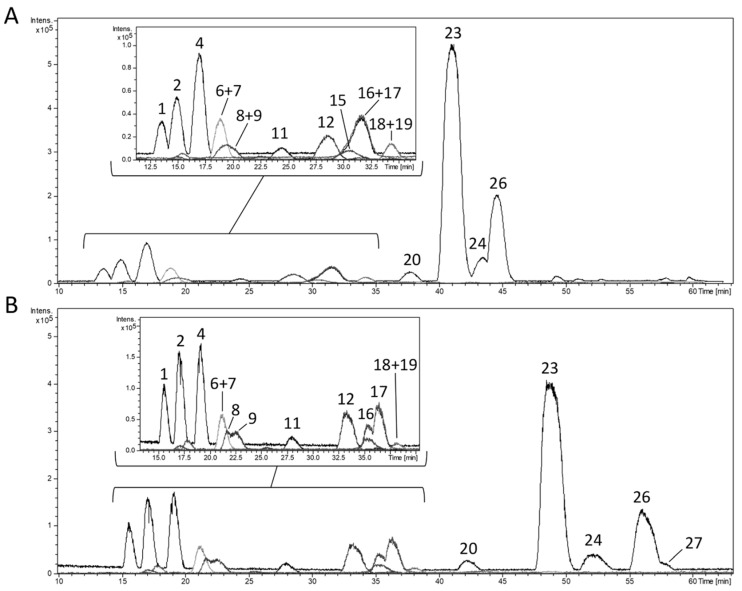
Base peak chromatogram (BPC) of a commercial lemon verbena extract (PLX^®^10) at 50 mg/mL obtained by semi-preparative HPLC-ESI-TOF-MS in negative ion mode applying (**A**) the initial multi-step lineal gradient from the analytical HPLC method, and (**B**) the final multi-step lineal gradient after optimization. Peak numbers correspond to those of Table 1.

In our study, a semi-preparative C18 (250 × 212 mm) column with 10 µm particle-size was used. The optimum solvents to be used as a mobile phase were based on the analytical chromatographic conditions maintaining the composition of the eluent A (acetic acid 0.5% in water) and B (methanol). Regular methods to isolate phenolics by HPLC used mainly gradient elution. An initial linear gradient time of 85 min and the flow-rate at 15 mL/min were used (Figure 2A). Then, the linear gradient was adapted to the new column characteristics to achieve the best separation results. The change of gradient affects the retention times and the chromatographic separation of the target compounds, so that testing of different experimental multi-step gradients was required. The best results were achieved with that detailed in the Materials and Methods section in comparison with other three different tested gradients. It is worth clarifying that the higher injected amount of extract influences the peak resolution as well. To increase the resolution among the peaks, the injected concentration was reduced from 75 to 50 mg/mL and the final flow rate used was 10 mL/min (Figure 2B). The final multi-step linear gradient allowed the separation of peaks 8–9, 16–17, 24, and 27 (according to the numeration of Table 1).

**Figure 3:**
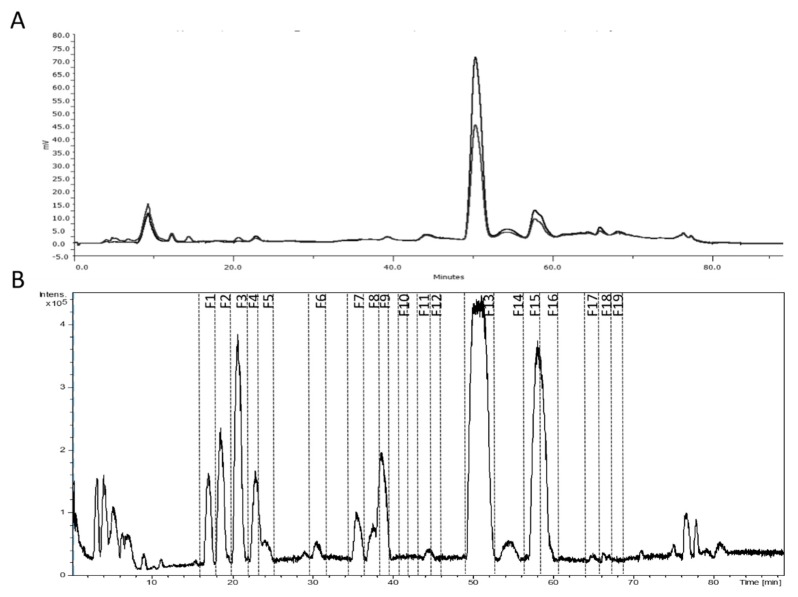
(**A**) UV chromatogram (240 and 280 nm) obtained by the semi-preparative HPLC system and (**B**) base peak chromatogram monitored by the ESI-TOF mass spectrometer, indicating the collected fractions from the commercial lemon verbena extract (PLX^®^10).

Afterwards, the collection of fractions was carried out taking into account the UV and MS information (Figure 3). The composition of the 19 obtained fractions was analyzed by the detailed HPLC-ESI-TOF-MS method (Table 2 and Supplementary Figure S2). Fractions were named as F1–F19 according to their elution order. The residue weight allowed the estimation of the relative amount of each compound(s). The major quantity of compound was obtained for verbascoside with 2.3 mg which suppose 9.2% of the injected amount of extract, close to the 10% declared by the manufacturer. The collected amount of some compounds that are not commercially available, as shanziside methyl ester or theveside, is worthy of consideration.

Three new compounds were detected only in isolated fractions due to their low concentration in the whole extract (Table 3). A new isomer of verbascoside, forsythoside A (*m/z* 623), was tentatively identified in fractions F13 and F15. Forsythoside A has been found in other *L. citriodora* extracts [17,38].

Fraction F18 contained not only martynoside but also another compound with the same *m/z* value. This peak was tentatively characterized as isomartynoside (*m/z* 651) as occurs in other *L*. *citriodora* work [50]. The presence of this compound in botanicals from the lamial species has been corroborated using NMR [51,52]. Isomartynoside was also detected in F19 where a third new compound was found. It was tentatively proposed as osmanthuside B (*m/z* 591) as it has been reported in a recent study [37].

### 3.3. Antioxidant Evaluation and Structure-Activity Relationships (SAR) of the Isolated Compounds

The antioxidant potential of natural extracts has attracted increased scientific interest due to its relationship with the prevention of chronic diseases. Nevertheless, the literature has reported a minor number of studies elucidating the antioxidant power of isolated compounds because of the necessary effort to obtain some of them.

In this context, three different in vitro methods were performed to explore the antioxidant capacity of the isolated compounds from lemon verbena and to establish structure-activity relationships (SAR). To the best of our knowledge, this is the first time that individual antioxidant activity of isolated compounds from lemon verbena has been tested. In addition, the antioxidant potential showed by the fractions was contrasted with the antioxidant activity of the whole extract.

**Figure 4:**
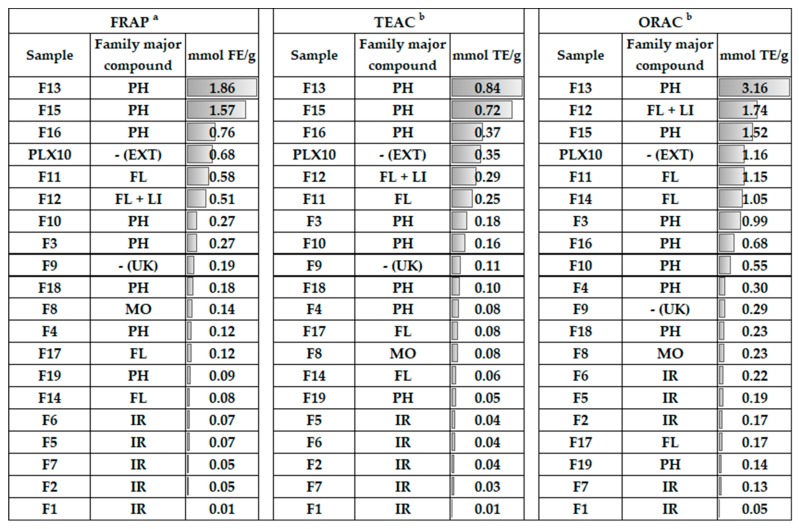
In vitro antioxidant activity estimated through ferric ion reducing antioxidant power (FRAP), trolox equivalent antioxidant capacity (TEAC), and oxygen radical absorbance capacity (ORAC) assays for the lemon verbena extract (PLX^®^10) and its collected fractions. Samples were indicated in decreasing order of activity for each assessed method including gradient data bars as graphical representation. ^a^ mmoles equivalents of Fe^2+/^g (dry weight), ^b^ mmoles equivalents de Trolox/g (dry weight), EXT: extract, FL: flavonoid, IR: iridoid, LI: lignan, MO: monoterpenoid, PH: phenylpropanoid, UK: unknown.

Regarding SET-based methods, the values for the whole lemon verbena extract were 0.676 ± 0.002 mmol FE/g in FRAP assay and 0.35 ± 0.03 mmol TE/g in TEAC assay (Figure 4 and Supplementary Table S1). Concerning isolated fractions, the FRAP values ranged from 0.009 ± 0.001 to 1.9 ± 0.1 mmol FE/g, while TEAC values were comprised from 0.008 ± 0.001 to 0.84 ± 0.04 mmol TE/g. In both assays, fraction F13 showed the highest antioxidant potential followed by F15, with greater values that those exhibited by the whole extract. Based on these experimental data, it could be hypothesized that verbascoside and isoverbascoside are the main responsible compounds of the antioxidant effects evidenced by lemon verbena extracts. In contrast, F1 exerted the minor antioxidant capacity and low values were obtained for F2 and F7 in both assays.

On the other hand, an ORAC assay was performed as the hydrogen-atom transfer based method and the whole extract showed an antioxidant activity of 1.2 ± 0.1 mmol TE/g. With regard to isolated fractions, the ORAC values varied from 0.051 ± 0.008 to 3.2 ± 0.3 mmol TE/g. Fraction F13 exhibited the strongest antioxidant power, as occurs in FRAP and TEAC assays but, in this case, was followed by F12. These two fractions and also F15 showed higher values than the whole extract. This reveals that luteolin-7-diglucuronide could play a relevant role in the antioxidant effects of lemon verbena as well. The lowest value was exhibited by F1 and fraction F7 exerted a weak antioxidant potential.

The evaluation of the antioxidant activity of the whole lemon verbena extract allowed its comparison with the literature. Arthur et al. evidenced that the FRAP value varies from 0.17 ± 0.05 to 0.73 ± 0.17 mmol FE/g depending on the part of the plant [53]. The FRAP value estimated in the present work is within that range. Furthermore, a lemon verbena extract containing 25% verbascoside showed a TEAC value 2.5-fold higher than our result, that is consistent with the 10% verbascoside in our extract. Concerning an ORAC assay, Buchwald-Werner et al. reported an antioxidant activity of 1.7 mmol TE/g using a commercial extract whose verbascoside content was not declared [54].

#### 3.3.1. Iridoids

This subclass of monoterpenoids possesses a six-membered ring, containing an oxygen atom, which is combined to a cyclopentane to form the iridane skeleton. Fractions containing an iridoid as major compound (F1, F2, F5, F6, F7) showed the lowest values in SET based methods. Some authors have demonstrated the weak antioxidant power of iridoids with respect to other families of phytocompounds concerning the SET mechanism [55,56]. It could be due to the absence of aromatic rings that allow electronic delocalization after losing an electron.

**Figure 5:**
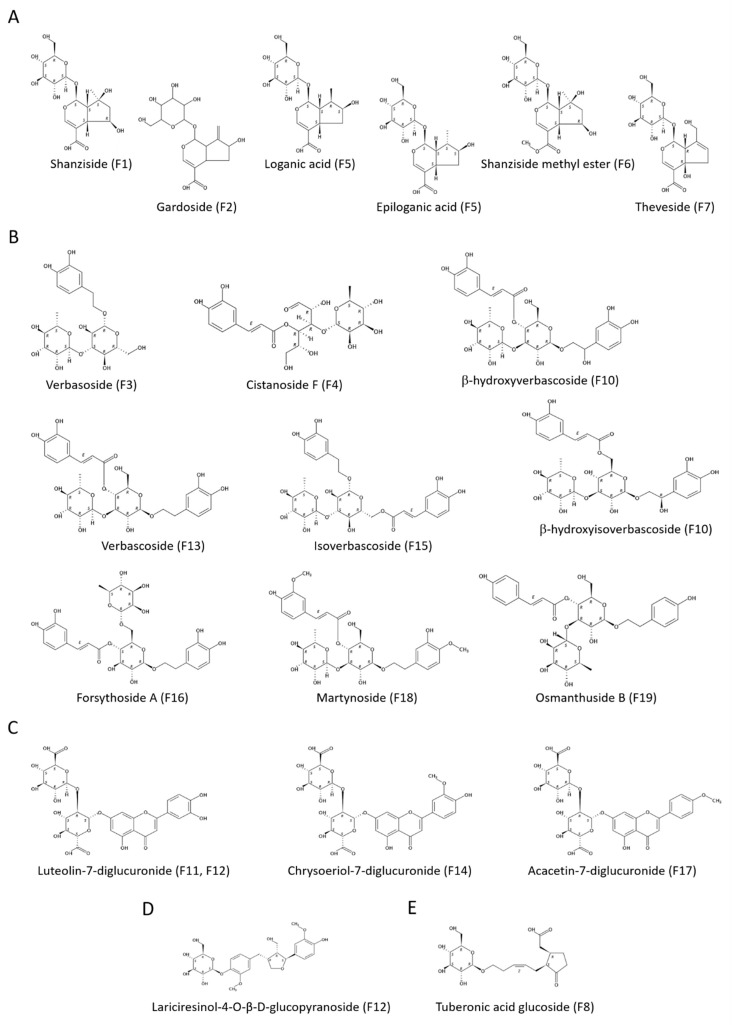
Chemical structures of the major compounds from the fractions classified in (**A**) iridoids, (**B**) glycosylated phenylpropanoids, (**C**) flavonoids, (**D**) lignan, and (**E**) monoterpenoid.

Among these fractions, F5 and F6 exhibited the highest values which were similar between them, while F2 and F7 showed similar weaker values and F1 exerted an insignificant antioxidant activity. Considering their common basic structure, the iridane skeleton, an iridoid could be partial or totally inactive with minor chemical modifications. It has been evidenced that antioxidant activity of iridoids do not improve with more hydroxyl groups (-OH), as occurs in polyphenols [57]. Loganic and epiloganic acids (F5) present one hydroxyl group less than shanziside (F1) and exhibited higher activity (Figure 5A). Nevertheless, shanziside esterification with a methyl group (F6) lead the strongest activity in the FRAP assay. Gardoside (F2) and theveside (F7) differs to shanziside with other substitutions and exerted higher antioxidant power.

Concerning the ORAC assay, it should be noted that the antioxidant power of fraction F2 was slightly higher than that exhibited by F7. The presence of ixoside in F2 could have contributed to the observed effect. This compound presents an additional carboxyl group (-COOH) where hydrogen atom is more reactive because of the carbonyl group (-C=O). However, the ability of fractions containing iridoids to reduce peroxyl radicals transferring a hydrogen atom was generally low in this assay. It could be due to the absence of aromatic rings that stabilize the resulting oxidized compound. Indeed, iridoids did not show a good hydrogen-donating ability in other studies [35,58].

#### 3.3.2. Glycosylated Phenylpropanoids

Phenylpropanoids belong to the phenolic compounds family which possess aromatic rings, giving more stable radical forms than other compounds after their oxidation. The influence of hydroxyl groups on the antioxidant capacity of other phenolic groups have been documented but, to our knowledge, this is the first study to establish SAR in phenylpropanoids. It was recognized that the catechol (*ortho*-dihydroxybenzene) unit is the main responsible structure in strong antioxidant phenolic compounds [59]. Fractions including glycosylated phenylpropanoids (F3, F4, F10, F13, F15, F16, F18, and F19) exhibited similar antioxidant power in FRAP and TEAC assays.

Fraction F13, corresponding to verbascoside, showed the strongest activity followed by F15 and F16 which presented its isomers isoverbascoside and forsythoside A, respectively. These three compounds are position isomers with two catechol units, one from each part connected to the glycosidic portion which are equal to hydroxytyrosol and caffeic acid (Figure 5B). Some authors have reported the higher antioxidant activity of verbascoside than that of isoverbascoside, reflecting the influence of the caffeoyl moiety position [60,61].

Verbasoside (F3) and β-hydroxyverbascoside together with β-hydroxyisoverbascoside (F10) exerted similar lower values. Verbasoside has one catechol unit so that its minor antioxidant activity is consistent. The extra hydroxylation in β-hydroxy(iso)verbascoside compared to (iso)verbascoside transform its phenylpropanoid part in a 3-4-dihydroxyphenylglycol moiety. There has been evidence of the stronger antioxidant activity of hydroxytyrosol than that exhibited by 3-4-dihydroxyphenylglycol [62].

Furthermore, F18 containing martynoside and isomartynoside showed a weaker antioxidant activity in both SET-based methods. There is not any catechol unit in these compounds where a hydroxyl group from each aromatic ring is etherified with a methyl group, forming a methoxy-phenylethanoid portion and a ferulic moiety. Ferulic acid has exhibited a lower antioxidant potential than caffeic acid in another study [63]. The major contribution of cistanoside F isomers in F4 lead to a lower antioxidant capacity of this fraction. Cistanoside F possesses only one phenolic ring in a caffeoyl moiety. The presence of two aromatic rings in (iso)martynoside could explain the higher activity of F18 than that of F4 in despite of the presence of caffeoyl moiety in cistanoside F. Nevertheless, it is worth to clarify the difference between this last compound and verbasoside: Both compounds present a catechol unit, but it is equal to hydroxytyrosol in verbasoside. The stronger antioxidant power of hydroxytyrosol compared to caffeic acid has been demonstrated [64]. Osmanthuside B (F19) possesses two monohydroxylated aromatic rings and exerts the lowest antioxidant power, corroborating the influence of the catechol group.

With respect to the ORAC results, there are some values that should be highlighted. Verbascoside (F13) showed the most powerful antioxidant effect with a value 2-fold higher than the estimated for isoverbascoside (F15), which in turn was 2-fold higher than that of forsythoside A (F16). Thus, the influence of the position of the caffeoyl group in verbascoside isomers was more notable for their capacity to transfer a hydrogen atom. Moreover, verbasoside (F3) exhibited a stronger antioxidant activity than of forsythoside A.

In contrast to SET-based methods, fraction F4 containing cistanoside F exerted a higher ORAC value than F18. In this case, the two hydroxyl groups in ortho position from the catechol unit could allow the formation of two hydrogen bonds with the two oxygen atoms of the peroxyl radical. This fact leads to a very compact reactant complex where the hydrogen abstraction would be more favorable as the quantum-mechanical tunneling explains [65].

#### 3.3.3. Flavonoids

Among the obtained fractions, four of them (F11, F12, F14, F17) presented a flavonoid as the major compound that belong to the flavones subgroup. Luteolin-7-diglucuronide was contained in fractions F11 and F12, but the determination of the composition of each fraction by HPLC-ESI-TOF-MS revealed the minor presence of lariciresinol-4-O-β-d-glucopyranoside in F12 and its contribution in the antioxidant effect was subject to study.

The SAR of the antioxidant potential of flavonoids was established by Rice-Evans et al. [63]. It is closely associated with the substitution with hydroxyl groups as occurs in other phenolic compounds, but the complexity of the flavonoid structure should be considered. Briefly, the radical-scavenging capacity is notably increased with a catechol unit in the B ring. A C2-C3 double bond configured with a 4-keto arrangement in the C ring, as is present in flavones, facilitates the electron delocalization from the B ring.

In SET-based methods, F11 and F12 showed the highest antioxidant activity. It was very similar between them and slightly lower than the exhibited by the whole extract. This is consistent with the luteolin-7-diglucuronide structure which possess the catechol unit in the B ring (Figure 5C). Estimated values for fraction F17 were 4-fold and 3-fold lower than F11/F12 in FRAP and TEAC assays, respectively. This fraction contained acacetin-7-diglucuronide where is a methoxy group in the B ring, so that the absence of the catechol group justifies the decreased antioxidant power. Chrysoeriol-7-diglucuronide (F14) exerted the lowest antioxidant power, but there were not large differences with F17.

Concerning the ORAC assay, it is worth remarking on the higher value determined for F12 than that of F11. In this case, lariciresinol-4-O-β-d-glucopyranoside seems to have a significant ability to scavenge peroxyl radicals transferring a hydrogen atom (Figure 5D). Furthermore, chrysoeriol-7-diglucuronide (F14) showed a higher antioxidant activity than acacetin-7-diglucuronide (F17). This is consistent with the absence of any hydroxyl group in the B ring of the last compound.

#### 3.3.4. Other Compounds

The remaining fractions (F8 and F9) exhibited a moderated antioxidant activity compared to other fractions. Fraction F8 was composed of tuberonic acid glucoside isomers and UK, while F9 contained UK, β-hydroxyverbascoside, and β-hydroxyisoverbascoside.

In all assays (FRAP, TEAC, and ORAC), F9 exerted a stronger antioxidant power than of F8. Ruberto and Barata analyzed the antioxidant activity of 100 terpenoids to establish SAR in this family of compounds [66]. In their study, it is related that the antioxidant potential of monoterpenoids tends to be higher with oxygenated substitutions in the following order: Phenol > allylic alcohol > aldehyde and ketone. Tuberonic acid is the 12-hydroxilated derivative of jasmonic acid which presents a keto group and a carboxyl group, so that it could have certain antioxidant activity (Figure 5E). Nevertheless, regarding the analytical composition of both fractions, UK seems to be the main contributor in the determined antioxidant activity for F8 that would be increased by β-hydroxyverbascoside and β-hydroxyisoverbascoside in F9. It is necessary to elucidate the chemical structure of UK to entirely establish the SAR for these compounds.

## 4. Conclusions

In the present work, a total of 30 compounds were identified in a commercial lemon extract by HPLC-ESI-TOF-MS previous to its fractionation by semi-preparative chromatography. The same powerful analytical technique was employed for analyzing the composition of the 19 fractions that were obtained and three new compounds were characterized. Afterwards, the antioxidant activity of the fractions was explored, and the SAR was established for the isolated compounds which were mainly iridoids, glycosylated phenylpropanoids, and flavonoids.

In conclusion, phenolic compounds exerted the strongest antioxidant power, as was expected. The presence of the catechol unit in those compounds is decisive, while our results demonstrated that hydroxyl groups are not fundamental in the iridoids subgroup of monoterpenoids. Nevertheless, fractions containing compounds from this last subgroup did not show an interesting antioxidant activity. Generally, the strongest antioxidant activity was exhibited by phenylpropanoids and, as far as we know, this is the first study where their SAR was explored. The influence of the position of the caffeoyl moiety in verbascoside and its isomers has been evidenced in our study and could be considered in the design and development of future bioactive compounds recovering processes. Moreover, verbasoside and β-hydroxy(iso)verbascoside showed an interesting antioxidant potential. Luteolin-7-diglucuronide exhibited the highest antioxidant potential among the studied flavonoids in all assays. The development of enriched extracts in these mentioned compounds, not only in verbascoside, could lead to greater antioxidant effects and improved functional ingredients for the prevention of chronic diseases.

## Figures and Tables

**Table 1:** Retention time (RT) and mass spectral data of the compounds characterized in the commercial lemon verbena extract (PLX^®^10) by reversed phase (RP)-HPLC-ESI-TOF-MS in negative mode.

Peak	RT (min)	[M-H]- Measured	[M-H]- Calculated	Error (ppm)	mSigma	Molecular Formula	Proposed Compound	Matrix
1	15.85	391.1236	391.1246	2.5	1.9	C 16 H 24 O11	Shanziside	*L. citriodora*
2	17.51	373.1122	373.114	4.9	3.1	C 16 H 22 O 10	Gardoside	*L. citriodora*
3	18.46	387.0934	387.0933	–0.3	5.7	C 16 H 20 O 11	Ixoside	*L. citriodora*
4	19.62	461.1654	461.1664	2.3	3.2	C 20 H 30 O 12	Verbasoside	*L. citriodora*
5	21.72	299.1116	299.1136	6.7	3.9	C 14 H 20 O 7	Salidroside	*S. viridis, E. rostkoviana, O. fragans*
6	22.14	487.1435	487.1457	4.6	1.6	C 21 H 28 O 13	Cistanoside F (isomer)	*L. citriodora*
7	22.22	487.1433	487.1457	5	4.1	C 21 H 28 O 13	Cistanoside F (isomer)	*L. citriodora*
8	22.46	375.1285	375.1297	3	4.3	C 16 H 24 O 10	Epiloganic acid	*L. graveolens*
9	23.33	375.1279	375.1297	4.8	3.9	C 16 H 24 O 10	Loganic acid	*L. citriodora*
10	23.71	373.1124	373.114	8.8	160.1	C 16 H 22 O 10	Secologanic acid	*L. graveolens*
11	27.74	405.139	405.1402	3	2.9	C 17 H 26 O 11	Shanziside methyl ester	*L. citriodora, L. alba*
12	30.28	389.1088	389.1089	0.3	3.1	C 16 H 22 O 11	Theveside	*L. citriodora*
13	31.29	489.1608	489.1614	1.1	3.3	C 21 H 30 O 13	Teucardoside	*L. citriodora, T. polium*
14	31.51	387.1654	387.1661	1.7	18.9	C 18 H 28 O 9	Tuberonic acid glucoside (isomer)	*L. citriodora*
15	31.76	445.2068	445.2079	2.5	24.6	C 21 H 34 O 10	Sacranoside A	*L. citriodora*
16	31.96	387.1654	387.1661	1.8	3.5	C 18 H 28 O 9	Tuberonic acid glucoside (isomer)	*L. citriodora*
17	32.69	387.2007	387.2024	4.6	1.4	C 19 H 32 O 8	UK	
18	33.93	639.1928	639.1931	0.5	30.6	C 29 H 36 O 16	b-hydroxy-(iso)-verbascoside	*L. citriodora*
19	34.25	639.1931	639.1931	0	41.7	C 29 H 36 O 16	b-hydroxy-(iso)-verbascoside	*L. citriodora*
20	36.36	637.1049	637.1046	–0.5	2.1	C 27 H 26 O 18	Luteolin-7-diglucuronide	*L. citriodora*
21	37.12	521.2016	521.2028	2.5	3.4	C 26 H 34 O 11	Lariciresinol-4-O-β-D-glucopyranoside	*L. graveolens*
22	40.85	621.1102	621.1097	–0.8	7	C 27 H 26 O 17	Apigenin-7-diglucuronide	*L. citriodora*
23	42.24	623.1999	623.1981	–2.9	3	C 29 H 36 O 15	Verbascoside	*L. citriodora*
24	43.33	651.1228	651.1203	–3.9	4.2	C 28 H 28 O 18	Chrysoeriol-7-diglucuronide	*L. citriodora*
25	45.29	621.1827	621.1766	–9.9	83.4	C 36 H 30 O 10	Diooflavone	*L. citriodora*
26	46.54	623.1988	623.1981	–1	3.3	C 29 H 36 O 15	Isoverbascoside	*L. citriodora*
27	47.24	637.215	637.2138	–2	5.2	C 30 H 38 O 15	Leucosceptoside A	*L. citriodora, L. alba, L. multiflora*
28	50.8	637.2142	637.2138	–0.6	4.8	C 30 H 38 O 15	Isoleucosceptoside A	*C. spinosum, Globularia* spp.
29	51.1	635.1271	635.1254	–2.7	6.1	C 28 H 28 O 17	Acacetin-7-diglucuronide	*L. citriodora*
30	54.63	651.2299	651.2294	–0.7	3.8	C 31 H 40 O 15	Martynoside	*L. citriodora*

**Table 2:** Collected fractions from a commercial lemon verbena extract (PLX^®^10) by semi-preparative chromatography, indicating the major compound(s) identified by HPLC-ESI-TOF-MS, the dry weight of the residue of each fraction and the relative amount taking account the injected quantity. Bold numbers correspond to the peaks with the highest intensity in each isolated fraction.

Fraction	Peak(s)	Major Compound(s)	Residue Weight (mg)	Relative Amount (%)
F1	**1**	Shanziside	0.7	2.8
F2	**2**, 3	Gardoside	1.0	4.0
F3	**4**	Verbasoside	0.9	3.6
F4	5, **6**, **7**, 8	Cistanoside F isomers	0.5	2.0
F5	**8**, **9**, 10	Loganic and epiloganic acids	0.9	3.6
F6	**11**	Shanziside methyl ester	0.6	2.4
F7	**12**	Theveside	0.6	2.4
F8	13, 14, 15, **16**, **17**	Tuberonic acid glucoside + UK	0.6	2.4
F9	**17**, 18, 19	UK	0.4	1.6
F10	17, **18, 19**	β-hydroxy(iso)verbascoside	0.4	1.6
F11	**20**	Luteolin-7-diglucuronide	0.5	2.0
F12	**20**, 21	Luteolin-7-diglucuronide	0.2	0.8
F13	22, **23**, 26, A	Verbascoside	2.3	9.2
F14	**24**, 25	Chrysoeriol-7-diglucuronide	0.9	3.6
F15	**26**, A	Isoverbascoside	1.1	4.4
F16	**A**, 27, 28	Forsythoside A	0.7	2.8
F17	**29**	Acacetin-7-diglucuronide	0.4	1.6
F18	**30**, B	Martynoside	0.7	2.8
F19	B, **C**	Osmanthuside B	0.3	1.2

**Table 3:** Retention time (RT) and mass spectral data of the compounds characterized only in fractions from the commercial lemon verbena extract (PLX^®^10) by RP-HPLC-ESI-TOF-MS in negative mode.

Peak	RT (min)	[M-H]- Measured	[M-H]- Calculated	Error (ppm)	mSigma	Molecular Formula	Proposed Compound	Reference	Matrix
A	49.84	623.1986	623.1981	–0.7	4.3	C 29 H 35 O 15	Forsythoside A	[17,38]	*L. citriodora*
B	56.59	651.2292	651.2294	0.3	5.1	C 31 H 40 O 15	Isomartynoside	[50,51,52]	*L. citriodora, P. carruthersii, S. tetradonta*
C	58.03	591.1972	591.2083	18.9	5.6	C 29 H 36 O 13	Osmanthuside B	[37]	*L. citriodora, C. tubulosa*

## Supplementary Materials

The following are available online at https://www.mdpi.com/2076-3921/8/8/324/s1, Figure S1. UV chromatograms at 280 nm and MS spectra for (A) peak 5 and (B) peak 28; Figure S2. Base peak chromatograms of the collected fractions from a commercial lemon verbena extract (PLX^®^10) and MS spectra of their major compound, including the peak numbers of Table 1; Table S1. *In vitro* antioxidant activity by FRAP, TEAC and ORAC assays for the commercial lemon verbena extract (PLX^®^10) and its collected fractions, expressed as the mean of three independent replicates ± the standard deviation.
