# Supplementary material for: Relationships Between Chemical Structure and Antioxidant Activity of Isolated Phytocompounds from Lemon Verbena
Source: Antioxidants (Basel). 2019 Aug 20;8(8):324. doi: 10.3390/antiox8080324 (PMC6719922; doi:10.3390/antiox8080324)
Supplement: Supplementary file 1 [file antioxidants-08-00324-s001.pdf]

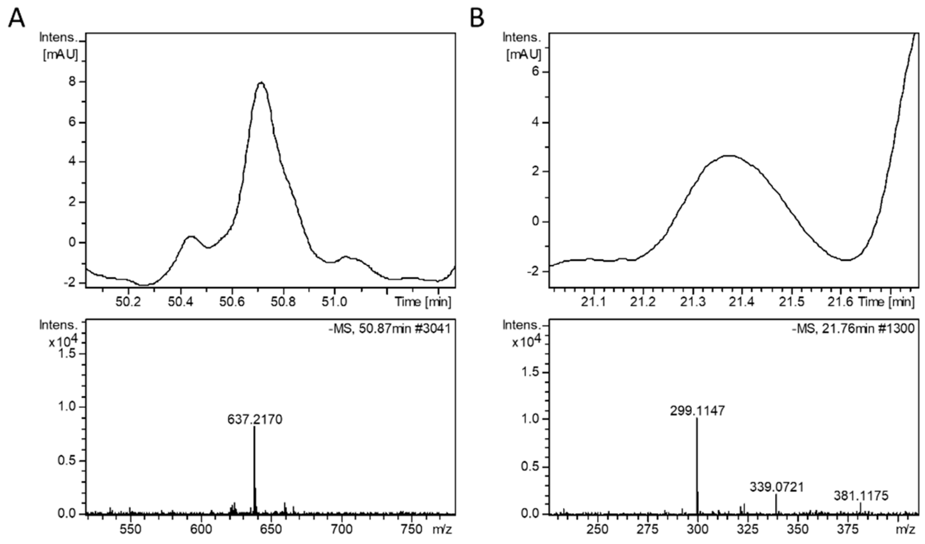

Figure S1. UV chromatograms at 280 nm and MS spectra for (A) peak 5 and (B) peak 28.

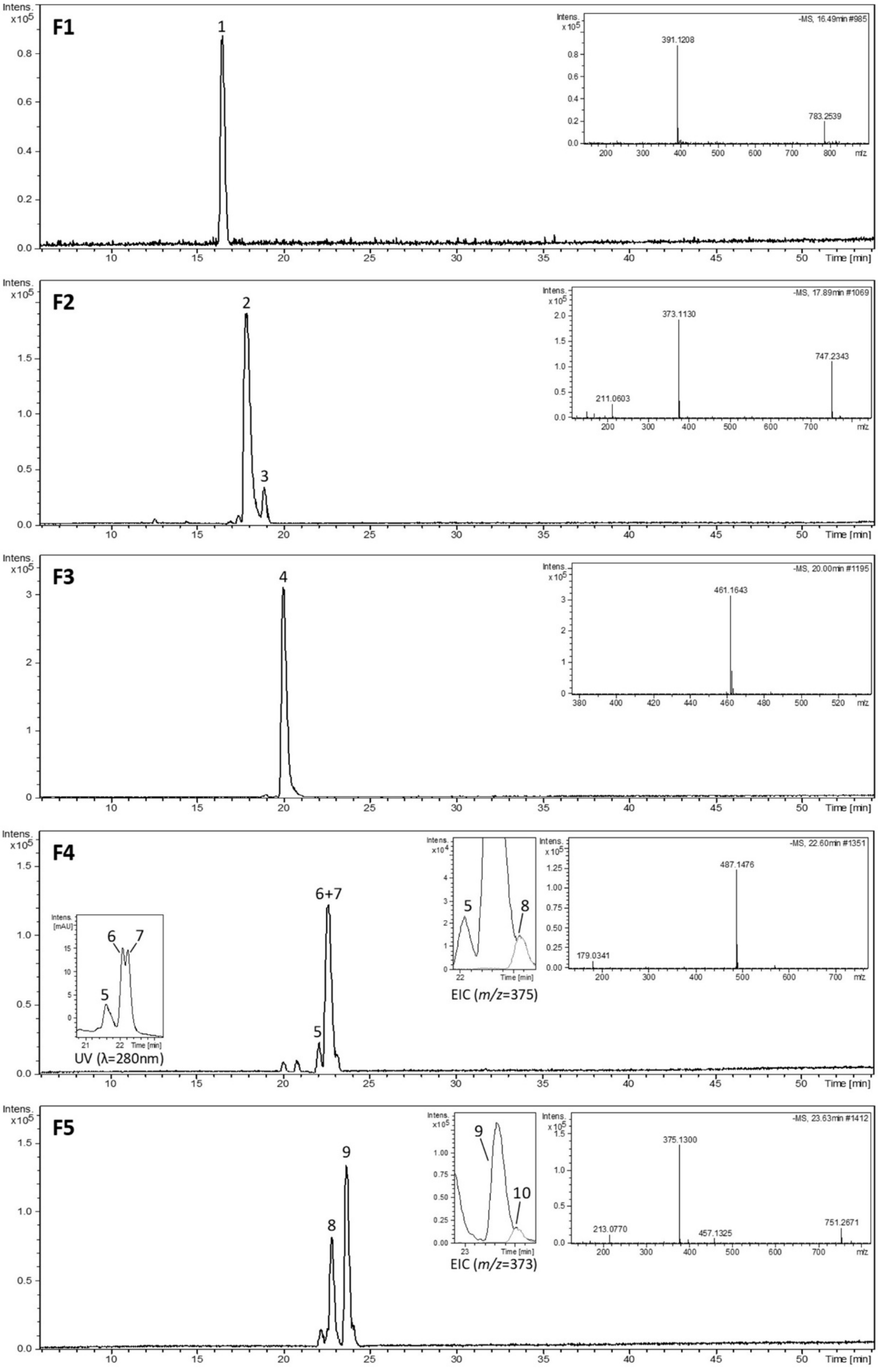

Figure S2. Base peak chromatograms of the collected fractions from a commercial lemon verbena extract (PLX®10) and MS spectra of their major compound, including the peak numbers of Table 1. UV/EIC chromatograms were add in those cases where they were considered necessary.

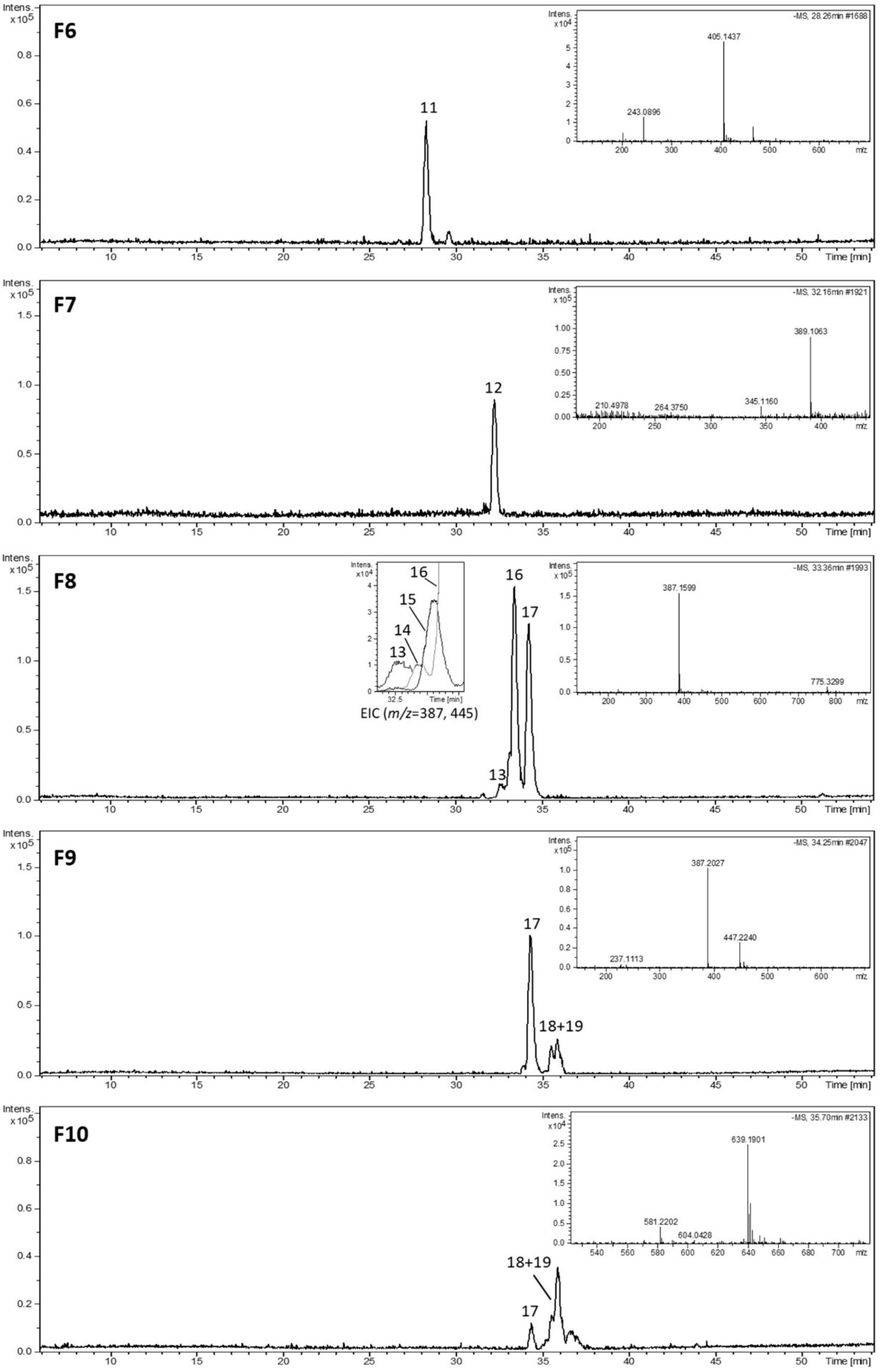

Figure S2. Base peak chromatograms of the collected fractions from a commercial lemon verbena extract (PLX®10) and MS spectra of their major compound, including the peak numbers of Table 1. UV/EIC chromatograms were add in those cases where they were considered necessary *(Continued)*.

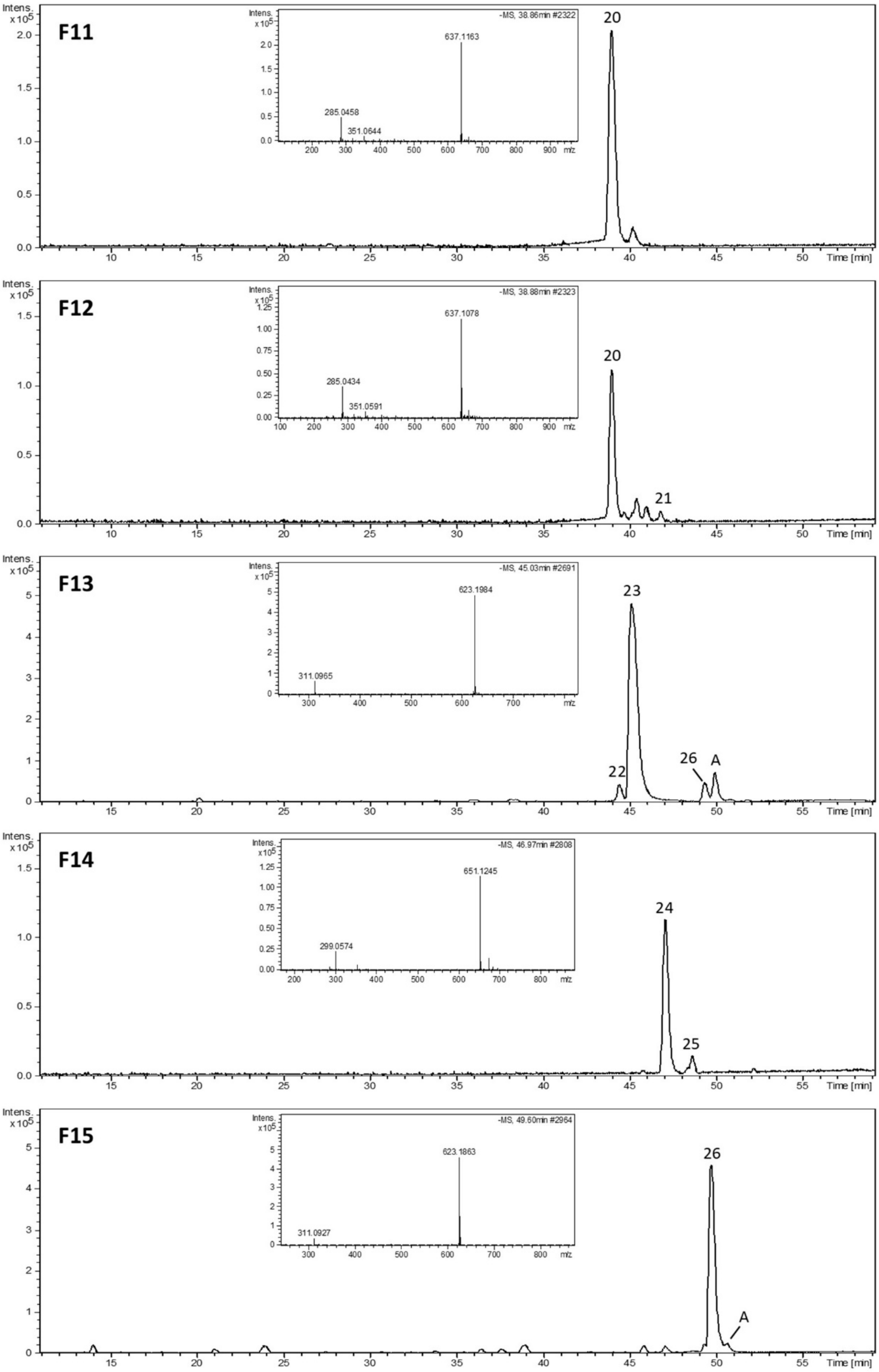

Figure S2. Base peak chromatograms of the collected fractions from a commercial lemon verbena extract (PLX®10) and MS spectra of their major compound, including the peak numbers of Table 1. UV/EIC chromatograms were add in those cases where they were considered necessary *(Continued)*.

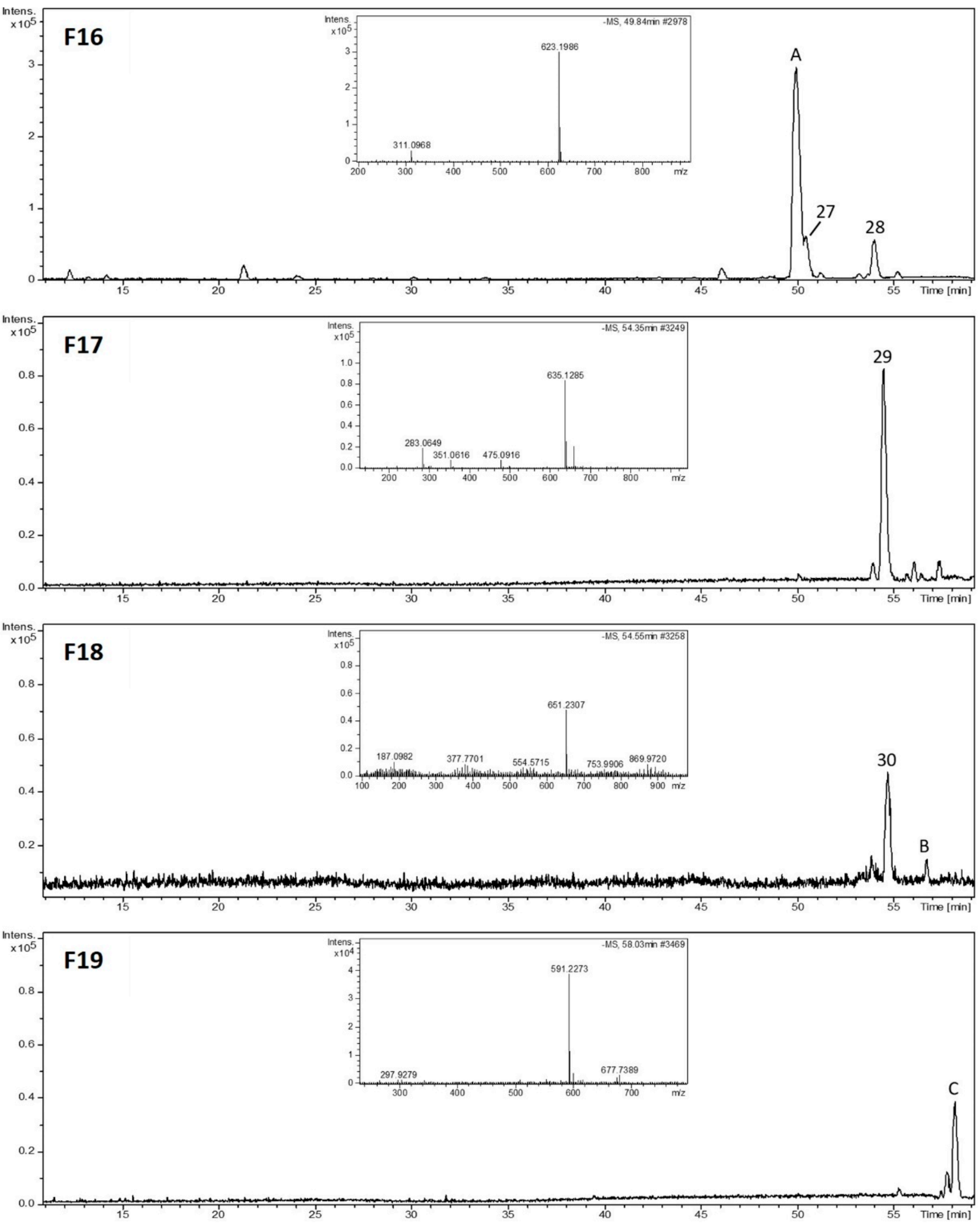

Figure S2. Base peak chromatograms of the collected fractions from a commercial lemon verbena extract (PLX®10) and MS spectra of their major compound, including the peak numbers of Table 1. UV/EIC chromatograms were add in those cases where they were considered necessary *(Continued)*.

Table S1. In vitro antioxidant activity by FRAP, TEAC, and ORAC assays for the commercial lemon verbena extract (PLX®10) and its collected fractions, expressed as the mean of three independent replicates ± the standard deviation. [a] mmoles equivalents of Fe^{2+}/g (dry weight), [b] mmoles equivalents of Trolox/g (dw).

Sample	FRAP[a]	TEAC[b]	ORAC[b]
**PLX®10**	0.676 ± 0.002	0.35 ± 0.03	1.2 ± 0.1
**F1**	0.009 ± 0.001	0.008 ± 0.001	0.051 ± 0.008
**F2**	0.045 ± 0.003	0.035 ± 0.001	0.174 ± 0.006
**F3**	0.270 ± 0.001	0.182 ± 0.004	0.99 ± 0.01
**F4**	0.122 ± 0.003	0.082 ± 0.001	0.30 ± 0.01
**F5**	0.068 ± 0.002	0.043 ± 0.003	0.19 ± 0.02
**F6**	0.074 ± 0.003	0.041 ± 0.009	0.223 ± 0.009
**F7**	0.048 ± 0.001	0.033 ± 0.003	0.131 ± 0.004
**F8**	0.137 ± 0.007	0.077 ± 0.007	0.23 ± 0.01
**F9**	0.192 ± 0.007	0.111 ± 0.002	0.290 ± 0.007
**F10**	0.27 ± 0.01	0.16 ± 0.01	0.55 ± 0.06
**F11**	0.58 ± 0.02	0.246 ± 0.003	1.2 ± 0.1
**F12**	0.51 ± 0.02	0.290 ± 0.001	1.74 ± 0.09
**F13**	1.9 ± 0.1	0.84 ± 0.04	3.2 ± 0.3
**F14**	0.084 ± 0.007	0.057 ± 0.006	1.05 ± 0.01
**F15**	1.57 ± 0.09	0.72 ± 0.04	1.5 ± 0.1
**F16**	0.83 ± 0.07	0.37 ± 0.02	0.68 ± 0.01
**F17**	0.120 ± 0.005	0.077 ± 0.006	0.169 ± 0.002
**F18**	0.179 ± 0.005	0.101 ± 0.02	0.23 ± 0.03
**F19**	0.091 ± 0.007	0.050 ± 0.005	0.12 ± 0.02
